# Annotations

**Published:** 1887-02-05

**Authors:** 


### ANNOTATIONS.

A Libel on Hospital Sunday.

Sir Edmund Currie, at the Mansion House on Tuesday, and not for the first time, gave utterance to a sentiment which is nothing but a libel upon Hospital Sunday as an institution of paramount importance to hospitals all the world over. He said that it had not been an unmixed blessing to the hospitals; meaning, presumably, that many people who formerly gave subscriptions to these charities, have discontinued their contributions to the hospitals. We beg leave to doubt this view of Hospital Sunday which Mr. Carr-Gomm very promptly proved wholly erroneous. The same idea finds expression in the report of the Committee of the Newcastle Infirmary, where a decrease in the annual subscriptions is attributed, without evidence to the institution of the Hospital Sunday Fund. We again canvass the view here expressed, and believe, with the late Canon Miller, the founder of Hospital Sunday, that giving is as much a matter of education as a knowledge of arithmetic, and that Hospital Sunday has been a great educator, and so an enormous benefactor to hospitals, quite apart from the money raised directly through its agency. We have thus far put our opinion only against that of others. The question at issue is of such vast importance that we think it well to give the facts also. In a provincial town, with a population more than double that of Newcastle, a similar contention was put to the test by contrasting the annual subscriptions received by all the medical charities in the year preceding the institution of Hospital Sunday with the current year's receipts. In the same way we have taken out the receipts from voluntary sources, of seventy-three London hospitals, in 1876, the year in which Hospital Sunday was first systematically organised in London, and in 1885. In both cases the figures prove that there has been a very considerable increase in the subscriptions to hospitals since the institution of Hospital Sunday. Thus, in the metropolis, the increase in the charitable revenue in the ten years amounts to no less than thirty-three per cent.; and although there are now eight per cent. more beds available than were provided for a similar population ten years ago, the free contributions of the people to the Metropolitan hospitals have increased by rather more than one penny per head per annum of the population, whilst the expenditure has only increased by about one farthing per head. The establishment of Hospital Sunday has, therefore, not only not caused a falling off in the subscription lists of the hospitals, but has produced an increase in their charitable revenue equal to three farthings per head of the population more than was received ten years ago,—that is, an increase of something like £20,000 a year in the total income of the seventy-three metropolitan hospitals. It is well that so manifest a libel should be exposed, and refuted by the publication of the facts here given.

The Home and the Hospital at Bristol.

Mr. Mark Whitwill, President of the Children's Hospital, Bristol, writes :—"I have read with much interest the article in your last number headed *From the Home to the Hospital*, and would venture to put before you the particulars of a simple plan that has been adopted in this city." The plan is this :—An ambulance is placed at the Central Police Station. When required for use it is horsed by the Fire Brigade. All the police stations, all the hospitals, and most of the cabstands are connected with the Telephone Exchange. From these points, or from the office or house of any subscriber to the Exchange, the ambulance can be immediately summoned, when an accident happens; the patient can be fetched from any part of the borough and removed immediately to the nearest hospital. The arrangement is sound to work easily and well. This plan offers a practical and highly economical solution of the ambulance question so far as regards towns which possess a Telephone Exchange and a Fire Brigade—that is to say, it is a solution of the question for almost all towns large enough to require the services of an ambulance. The carriage no longer requires to be either housed or horsed if the police and the firemen can be brought to sympathise with the movement. Thus two of the heaviest items of expenditure are at once disposed of. Mr. Whitwill does not state whether the ambulance is also manned by the police or the men of the fire brigade; but we should think the latter might very well be made available. Suggestions bearing on this important topic will be welcomed from any quarter.

---

Lord Lanesborough, whose "country house" stood on the site now occupied by St. George's Hospital, used to regard dancing as a universal panacea. When Queen Anne lost her consort, he actually went so far as to persuade her that, if she would but take to his favourite exercise, it would assuage her grief. We are not inclined to go quite the length of the assertion of the "dancing lord," but, if dancing will not directly cure our ills, it can be made indirectly to assist in that end. The annual ball in aid of the Nottingham Children's Hospital invariably results in a substantial sum being handed over to that excellent institution, and this year it has proved no exception to the rule, both from a pecuniary and from a terpsichorean point of view, nearly 450 tickets being sold. From 7 p.m. to 2 a.m. all went "merry as a marriage bell," thanks to the excellent arrangements of Mr. Bernard Wilcockson, the hon. secretary. At intervals the gas was lowered and the room bathed in artificial lights, producing a scene of exquisite beauty. The proceeds amounted to over £100.

